# Alpha-1 antitrypsin regulates neutrophil reactive oxygen species production via inhibition of key players of the respiratory burst oxidase system

**DOI:** 10.1186/1753-6561-7-S1-P7

**Published:** 2013-01-30

**Authors:** Bader Alfawaz, DA Bergin, Noel G McElvaney, EP Reeves

**Affiliations:** 1 Department of Medicine, Beaumont Hospital, Royal College of Surgeons, Dublin, Ireland

## Background

Activation of neutrophils sequestered in the alveolar milieu can cause the release of reactive oxygen species (ROS), increasingly regarded as key substances modulating epithelium dysfunction and disruption. These oxidants are generated by the neutrophil respiratory burst oxidase system that reduces molecular oxygen (O2) to superoxide (O2-). Alpha-1 antitrypsin (AAT) deficiency (AATD) provides us with the most definitive evidence for the physiological and clinical importance of AAT and in this study we examined the immunomodulatory activity of AAT and investigated whether neutrophil ROS production was regulated by AAT.

## Methods

Neutrophil O2- production in response to fMLP (10-6M) and IL-8 (10ng) was measured by a cytochrome C reduction assays respectively. Analysis of ERK phosphorylation in response to fMLP ± AAT was carried via Western blot analysis of neutrophil whole cell lysates. To verify fMLPs interaction with its cognate receptors fMLP R1 (FPR1) and fMLP R2 (FPR2), flow cytometry was used to measure levels of FITC labeled fMLP binding to the membranes of neutrophil.

## Results

In this study we demonstrate using in vitro models that AAT modulates neutrophil O2- production elicited by fMLP and IL-8 in a dose dependant manner (P<0.05). Mechanisms of inhibition were investigated and in vitro studies revealed that AAT functions to inhibit fMLP signaling through inhibiting its interaction with its receptors FPR1 and FPR2 on the neutrophil plasma membrane.

## Conclusions

The potential of AAT as a regulator of neutrophil ROS production adds a new understanding to the role of AAT in health and disease.

